# FGF2 Delays Tectal Neurogenesis, Increases Tectal Cell Numbers, and Alters Tectal Lamination in Embryonic Chicks

**DOI:** 10.1371/journal.pone.0079949

**Published:** 2013-11-12

**Authors:** Luke D. McGowan, Roula A. Alaama, Georg F. Striedter

**Affiliations:** Department of Neurobiology and Behavior, Center for the Neurobiology of Learning and Memory, University of California Irvine, Irvine, California, United States of America; Universidade Federal do ABC, Brazil

## Abstract

Intraventricular injections of the fibroblast growth factor 2 (FGF2) are known to increase the size of the optic tectum in embryonic chicks. Here we show that this increase in tectum size is due to a delay in tectal neurogenesis, which by definition extends the proliferation of tectal progenitors. Specifically, we use cumulative labeling with the thymidine analog EdU to demonstrate that FGF2 treatment on embryonic day 4 (ED4) reduces the proportion and absolute number of unlabeled cells in the rostroventral tectum when EdU infusions are begun on ED5, as one would expect if FGF2 retards tectal neurogenesis. We also examined FGF2′s effect on neurogenesis in the caudodorsal tectum, which is born 2-3 days after the rostroventral tectum, by combining FGF2 treatment on ED4 with EDU infusions beginning on ED8. Again, FGF2 treatment reduced the proportion and number of EdU-negative (i.e., unlabeled) cells, consistent with a delay in neurogenesis. Collectively, these data indicate FGF2 in embryonic chicks delays neurogenesis throughout much of the tectum and continues to do so for several days after the FGF2 injection. One effect of this delay in neurogenesis is that tectal cell numbers more than double. In addition, tectal laminae that are born early in development become abnormally thin and cell-sparse after FGF2 treatment, whereas late-born layers remain unaffected. Combined with the results of prior work, these data indicate that FGF2 delays tectal neurogenesis and, thereby, triggers a cascade of changes in tectum size and morphology.

## Introduction

Comparative work in evo-devo neurobiology has shown that evolutionary increases in brain region volumes are often due to delays in cell cycle exit of neuronal precursors [1], [2]. By delaying cell cycle exit, the period of progenitor proliferation is extended, which increases the progenitor pool and, other things being equal, adult cell population size. In a previous study [3], we reported that injections of fibroblast growth factor-2 (FGF2) into the cerebral ventricles of embryonic chicks increases the volume and surface area of the optic tectum. We used FGF2 because it has been reported to delay neurogenesis in some mammalian neural progenitors [4]. However, it had not been shown to regulate the timing of neurogenesis in the avian optic tectum. In our previous study, we provided indirect evidence suggesting that the FGF2-induced increase in tectum size is caused by a delay in tectal neurogenesis, but we had not directly demonstrated this delay. In the present paper, we show for the first time that FGF2 injections delay cell cycle exit in chick tectal progenitors and, consequently, increase the absolute number of tectal cells. We also show that FGF2 delays neurogenesis for several days after the FGF2 injections and that it changes the birthdates and thickness of some tectal laminae.

**Figure 1:**
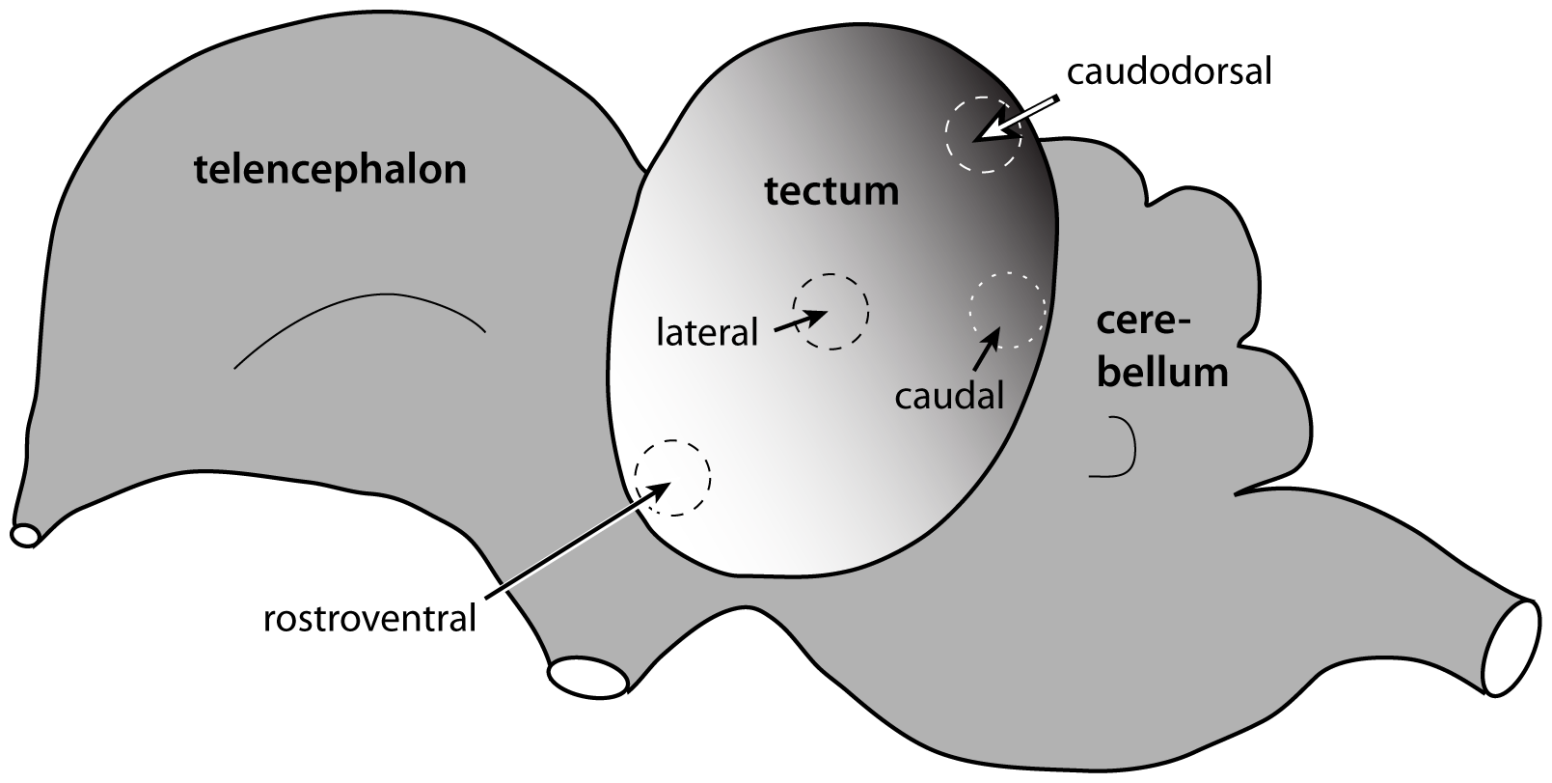
There is a neuronal birthdate gradient within the chick optic tectum. Previous birthdating studies [5] revealed a lateral rostroventral to medial caudodorsal birthdate gradient within the optic tectum. The lateral to medial dimension is not represented here. Taking advantage of this birthdate gradient, we looked for FGF2-induced differences in cell cycle exit in the rostroventral tectum at the beginning of tectal neurogenesis and in the caudodorsal tectum at the end of tectal neurogenesis.

Our experimental approach builds on a previous study of tectal neurogenesis in chicks by Lavail and Cowan [5], who used a “cumulative labeling” method to establish the time of origin of neurons throughout the tectum. Starting at various stages of embryonic development, they exposed chicks to tritiated thymidine, which is incorporated into the DNA of proliferating cells as they pass through S-phase. Because the tritiated thymidine remains continuously available within the egg, all cells born (i.e., undergoing terminal mitosis) after the tritiated thymidine injection will become radioactive (i.e., labeled). In contrast, all the *unlabeled* cells must have been born prior to the thymidine injection. By examining birds that were injected with tritiated thymidine at different stages of embryonic development, Lavail and Cowan were able to deduce cellular birthdates across the different parts of the tectum. They reported a rostroventral to caudodorsal gradient, with tectal neurogenesis beginning in the lateral rostroventral region as early as ED3 and ending in the medial caudodorsal region around ED9 (Fig. 1). Lavail and Cowan also determined that the middle tectal layers were born after the superficial laminae, which were, in turn, born after the deep layers.

**Figure 2:**
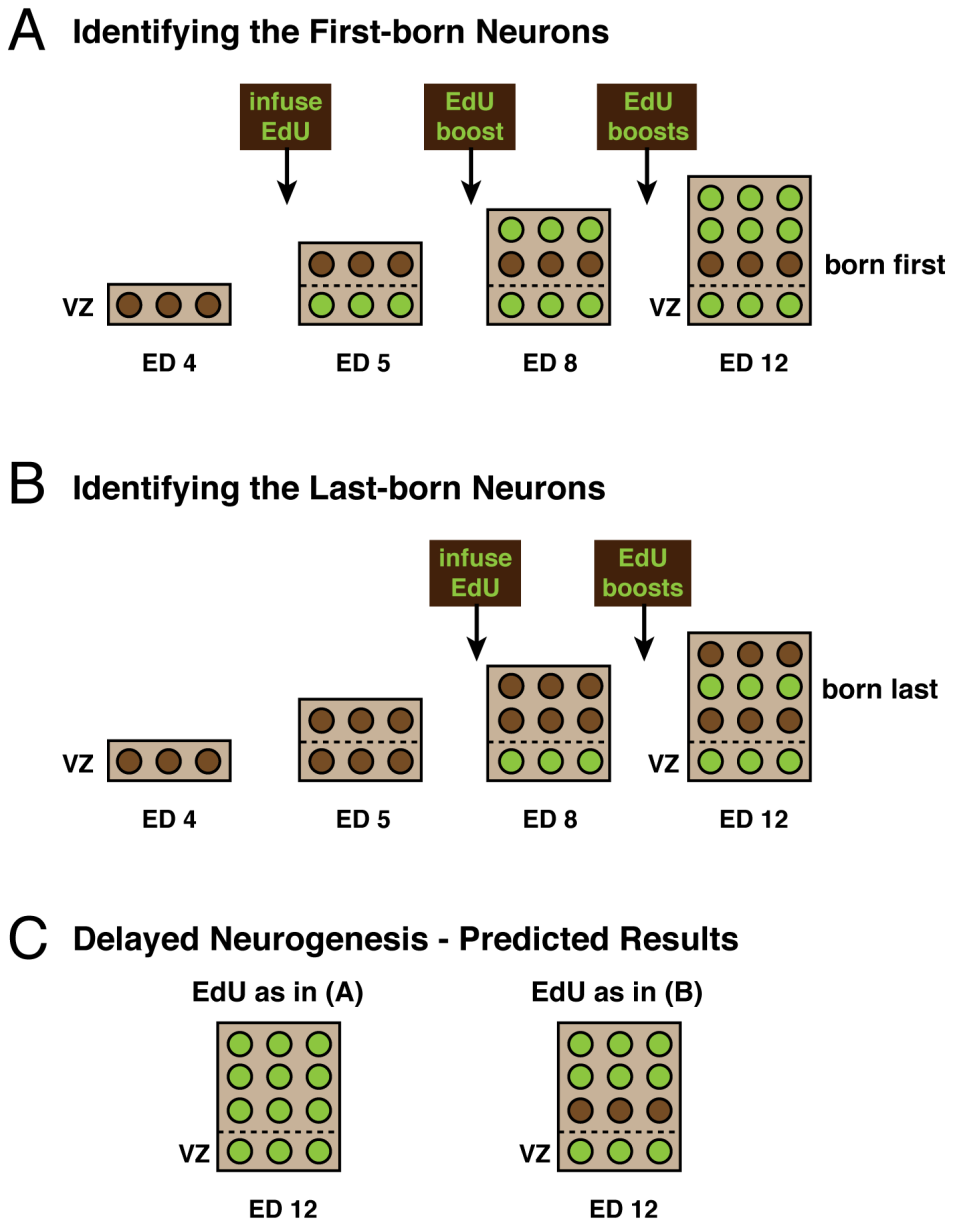
Schematic of the experimental design and predictions. On either ED5 (A) or ED8 (B), control and FGF2-treated embryos were infused with EdU followed by regular booster shots sufficient to saturate the system. Birds were then sacrificed on ED12 for processing. EdU is taken up by all proliferating cells as they pass through S-phase (shown in green), whereas all cells born prior to infusion are EdU*-unlabeled* (shown in brown). In the ED5 condition (A), the early born neurons in the deep layers of control tecta have already been born at the time of EdU infusion and so do not take up EdU (brown). In contrast, if FGF2 delays neurogenesis, then all cells should take up the EdU (green) in the treated embryos (C), because no neurons would have been born at the time of EdU infusion. In the ED8 condition (B), only late born neurons in the middle layers of control tecta have yet to be born, and so take up the EdU (green). If FGF2 delays tectal neurogenesis, then one would expect the neurons in the most superficial layers also to incorporated the EdU, because they, too, would not yet have been born before the EdU infusion (C). The ventricular zone (VZ) contains proliferating cells and is, therefore, EdU-positive after the EdU infusions have begun.

In the present study, we injected the thymidine analog EdU (5′-ethynyl-2′-deoxyuridine) rather than tritiated thymidine, because it can be detected more easily. As expected, we confirmed the neurogenetic gradient reported by Lavail and Cowan [5], though the absolute timing of tectal neurogenesis onset and offset in our chicks are more in line with those reported by Fujita [6]. More important, we then used the cumulative EdU labeling method to examine the effects of FGF2 on neurogenesis timing in the optic tectum. A delay in neurogenesis should decrease the number of EdU-*unlabeled* cells throughout the tectum, but those changes should be easiest to detect in the early-born rostroventral tectum and the late-born caudodorsal tectum (Fig. 2). Specifically, if EdU is injected shortly after the normal onset of tectal neurogenesis (embryonic day 5), then the rostroventral tectum should contain a relatively small number of EdU-unlabeled cells in control embryos. However, if FGF2 delays neurogenesis, then that number should fall to zero in the FGF2-treated embryos. In contrast, if EdU is injected shortly before the end of normal tectal neurogenesis (embryonic day 8), then the caudodorsal tectum in control embryos should contain mostly EdU-unlabelled cells (i.e., very few labeled cells); if FGF2 delays neurogenesis, then this region of the tectum should contain significantly fewer EdU-unlabeled cells (i.e., both proportionately and absolutely more labeled cells) in the FGF2-treated embryos. In other words, we focused on the rostroventral and caudodorsal tectum because in these regions the contrast between FGF2 -treated and control embryos (in terms of EdU-labeled versus unlabeled cells) should be greatest.

In this study we also analyze FGF2-induced changes in the morphology of tectal laminae, including changes in laminar thickness and the number of cells above a unit area of ventricular zone surface. We focus these analyses on changes in the lateral and caudal areas of the tectum, as these areas correspond to regions where FGF2-induced laminar disturbances had been observed in our previous study [3]. Finally, we provide a quantitative analysis of FGF2-induced changes in the birthdates of tectal laminae, which give us clues about how delays in neurogenesis affect cell migration and, perhaps, cell type specification [2], [7], [8].

What remains unclear from all this work is why the FGF2 effect in birds appears to be limited to the optic tectum, whereas it extends into the telencephalon in mammals [4]. Presumably, this species difference relates to the relatively low levels of FGF receptor expression in the chick telencephalon [9].

## Materials and Methods

This study did not require approval of UC Irvine's Institutional Animal Care and Use Committee, because embryos are not covered by the relevant legislation. Fertile chicken eggs (*Gallus gallus domesticus*) were obtained from a commercial supplier and incubated in a rotating egg incubator (PROFI-I, Lyon Technologies, Chula Vista, CA) at 38° and 50–60% humidity. On embryonic day 4 (ED4), 0.5–1 µl of human recombinant bFGF (a.k.a. FGF2; R&D Systems, Minneapolis, MN; 100 ng/ µl, dissolved in 0.1 M phosphate buffered saline (PBS) and dyed with methylene blue) was injected into the tectal ventricles. The injected FGF2 rapidly diffused throughout the ventricles, regardless of injection site. Control chicks were injected with 0.5–1 µl of dyed 0.1 M PBS. After injection, the eggs were resealed and transferred to the incubator.

Cumulative labeling was then performed as illustrated in Figure 2. On either ED5 or ED8, control and FGF2-treated embryos were exposed to 20 µl EdU (Click-iT EdU kit, Cat. #C10337, Invitrogen, Carlsbad, CA; 2.5 µg/µl, dissolved in 0.1 M PBS). Booster shots of EdU (20 µl) were given every 48 hours (if starting on ED5) or every 24 hours (if starting on ED8). These booster shots were experimentally determined to be sufficient to achieve saturation labeling of cells born after the EdU infusions were begun, without noticeably affecting development or survival. All birds were sacrificed on ED12 for processing.

The embryos were immersion-fixed overnight in methacarn (60% methanol, 30% chloroform, 10% glacial acetic acid), dehydrated, embedded in paraffin, and sectioned at 8 µm. Roughly 20–30 evenly spaced sections from throughout the tectum were mounted onto Superfrost Plus slides (Fisher Scientific), stained with Giemsa (Sigma-Aldrich Inc.; St. Louis, MO), and cover-slipped. Based on examination of these Giemsa-stained sections, we identified specific tectal sectors in individual birds (Fig. 1). For simplicity, we refer to the lateral rostroventral tectum as “rostroventral” tectum and to the medial caudodorsal tectum as “caudodorsal” tectum. Additional sections from these two tectal sectors were mounted, dewaxed, and prepared for EdU processing.

Our EdU processing followed the protocol of Warren et al. [10]. The tissue was permeabilized with 0.5% Triton X-100/PBS before being exposed to the EdU reaction cocktail for 30 minutes (reaction buffer, CuSO4, Alexa Fluor 488 azide, and buffer additive as per manufacturer's protocol; Click-iT EdU kit, Cat. #C10337, Invitrogen). After washing with PBS, slides were cover-slipped for photography and analysis.

All cell counts and laminar thickness measurements were made in ImageJ (NIH, Bethesda, MD). Thickness measurements were performed on Giemsa-stained sections in lateral and caudal areas of the tectum (corresponding to regions containing FGF2-induced “volcanoes” and folds, respectively; see [3]). The histological borders of the deep, middle and superficial laminae were readily distinguishable in Giemsa stains based mainly on cell density differences (Fig. 6A). Our deep, middle and superficial laminae correspond to what Lavail and Cowan [5] refer to as Zone 1 (including the stratum griseum centrale), Zone 3 (including cell-dense lamina (i) of the stratum griseum et fibrosum superficiale (SGFS)), and Zone 2 (including the cell-dense layers in lamina g of the SGFS), respectively. Lamina thickness was estimated by measuring the area of individual laminae along a 250 µm stretch of tectum (measured tangentially) and then dividing the areas by the length.

**Figure 3:**
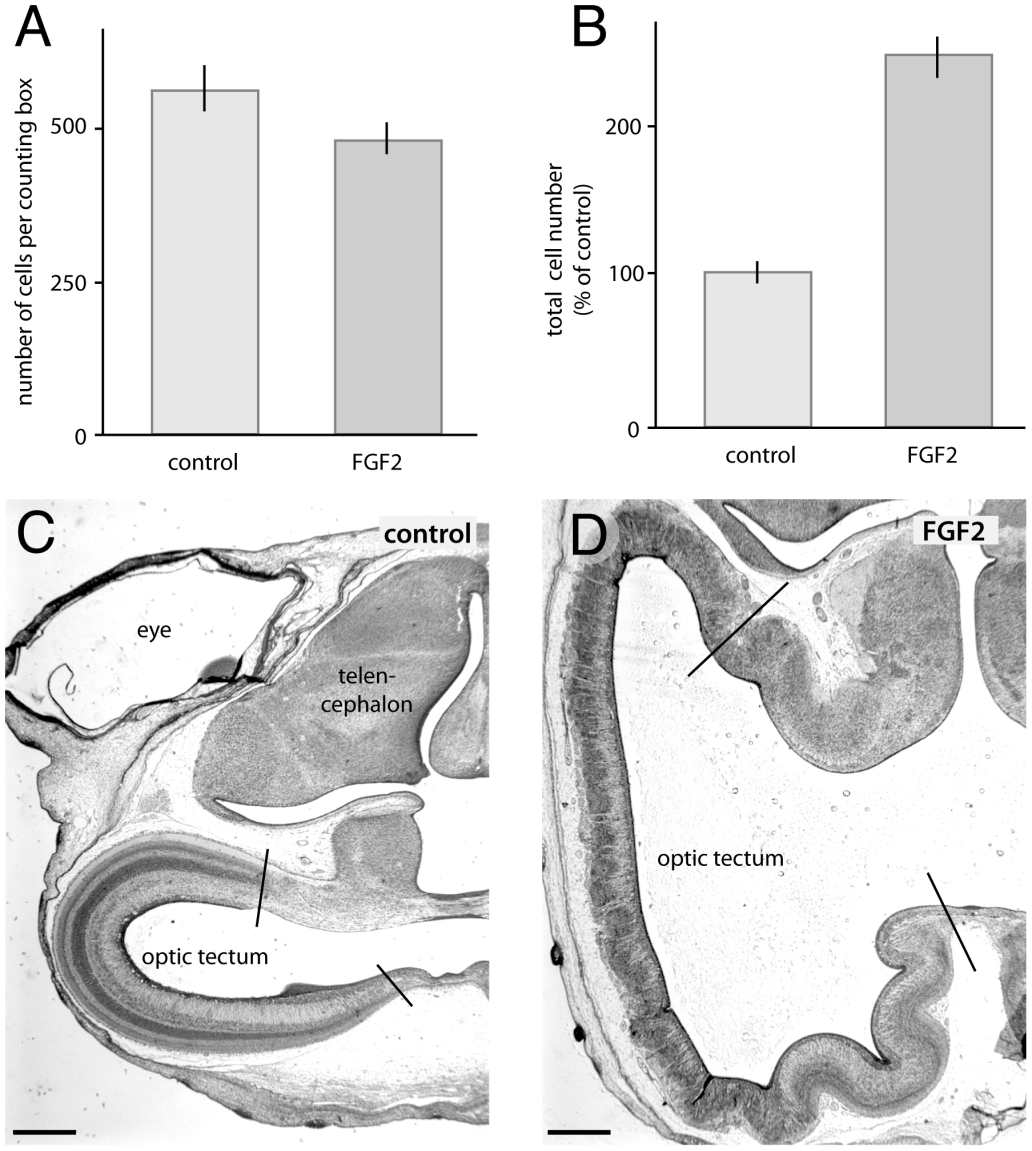
FGF2 treatment increases the absolute number of tectal cells. Intra-ventricular injections of FGF2 on ED4 decrease the number of cells above a unit area of ventricular surface by 14% compared to controls (A). Taking into account our previous observation that FGF2 increases tectal ventricular surface area by 181% [3], we estimate that FGF2 treatment increases total tectal cell numbers by approximately 140% compared to controls (B). The photographs (C and D) illustrate the dramatic tangential expansion of the tectum in FGF2 treated birds, relative to controls. Scale bar = 1 mm.

**Figure 4:**
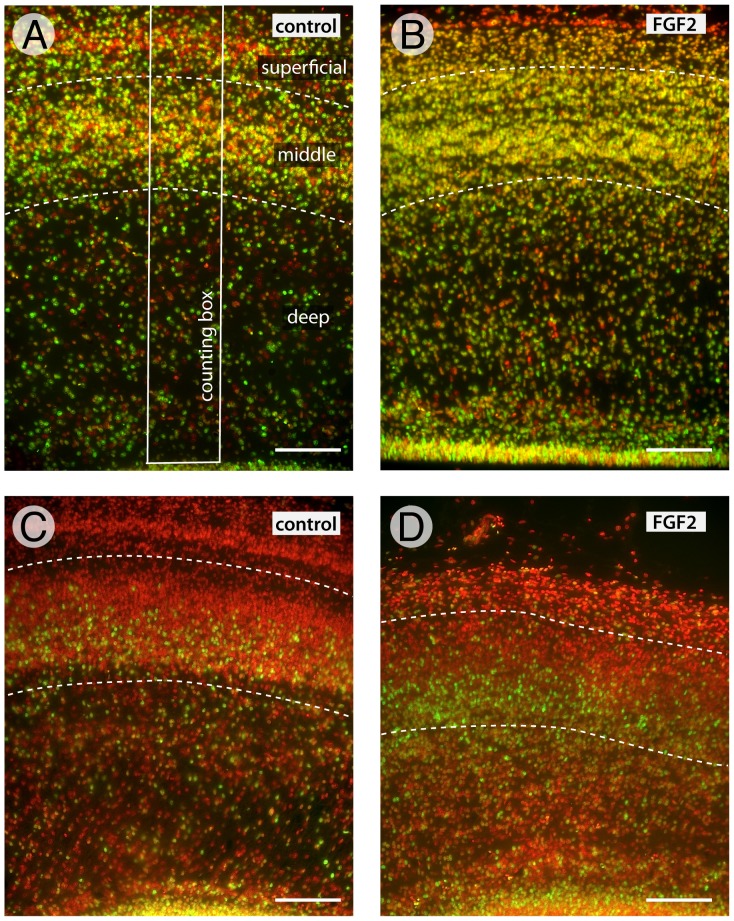
Sample data showing that FGF2 treatment delays tectal neurogenesis. Using counting boxes that span the radial extent of the tectum (white rectangle in A), we counted the total number of cells (bisbenzimide counterstain shown in red) and the number EdU-positive cells (Alexa Fluor 488 appears yellow) in the deep, middle and superficial layers. Cumulative EdU labeling beginning at ED5 (A and B) revealed fewer unlabeled (EdU-negative) cells in the rostroventral tectum of FGF2 treated birds than in controls (quantitative comparisons are shown in Fig. 5 A, B), which means that fewer cells had exited the cell cycle by ED5. Similarly, cumulative EdU labeling beginning at ED8 (C and D) revealed fewer EdU-unlabeled cells in the caudodorsal tectum of FGF2-treated birds than in controls (quantitative comparisons are shown in Fig. 5 C, D.). Scale bar = 100 µm.

Cell counts were made from EdU stained sections counterstained with bisbenzimide. As noted earlier (Fig. 2), cell counts of EdU labeled and unlabeled cells were performed in either the rostroventral (ED5) or caudodorsal (ED8) parts of the tectum. To estimate total tectal cell numbers we counted, in addition, sample regions the lateral and caudal parts of the tectum, averaging the counts across all areas before averaging across embryos. When counting cells in individual laminae, those laminae were demarcated in bisbenzimide-stained sections and, when necessary, neighboring Giemsa stained sections. Cell counts were performed in ImageJ using a counting box that extended from a unit area of ventricular zone surface (8 µm thick by 100 µm long; Fig 4A) to the tectal surface. These columnar counting boxes allowed us to measure cell density and EdU labeling across the tectal laminae in FGF2 treated and control embryos. All statistical tests were performed in the program JMP 10 (SAS, Cary, N.C., USA).

## Results

We first report how FGF2 treatment affects total cell numbers in the tectum by ED12. This is followed by analyses of FGF2-induced changes in the onset and offset of tectal neurogenesis. Next, we report on FGF2-induced changes in the thickness and cell density of tectal laminae. Finally, we describe how FGF2 changes the birthdates of selected tectal laminae.

### FGF2 leads to an increase in cell numbers in the tectum

Since delaying cell cycle exit causes progenitors to undergo additional rounds of cell division, one would expect an FGF2-induced delay in neurogenesis to increase total tectal cell numbers (rates of cell death and other factors being equal). To test this prediction, we measured the number of cells radially above a unit area of ventricular zone surface (8 µm section thickness×100 µm) and found it to be 14% lower in FGF2 treated birds than in controls (Fig. 3; t(19) = −1.83; p<0.05; n = 10 controls, 11 FGF2-treated embryos). At first blush, these data suggest that FGF2 treatment decreases tectal cell numbers. However, as illustrated in Fig. 3C,D and quantified in our earlier study [3], FGF2 treatment also increases the tectum's ventricular surface area by 181%, almost tripling its size. Accounting for this tangential expansion, we estimate that FGF2-treated birds have 140% more tectal cells than controls (Fig. 3B; t(19) = 9.70; p<0.0001; n = 10, 11).

### FGF2 treatment delays the onset of tectal neurogenesis

**Figure 5:**
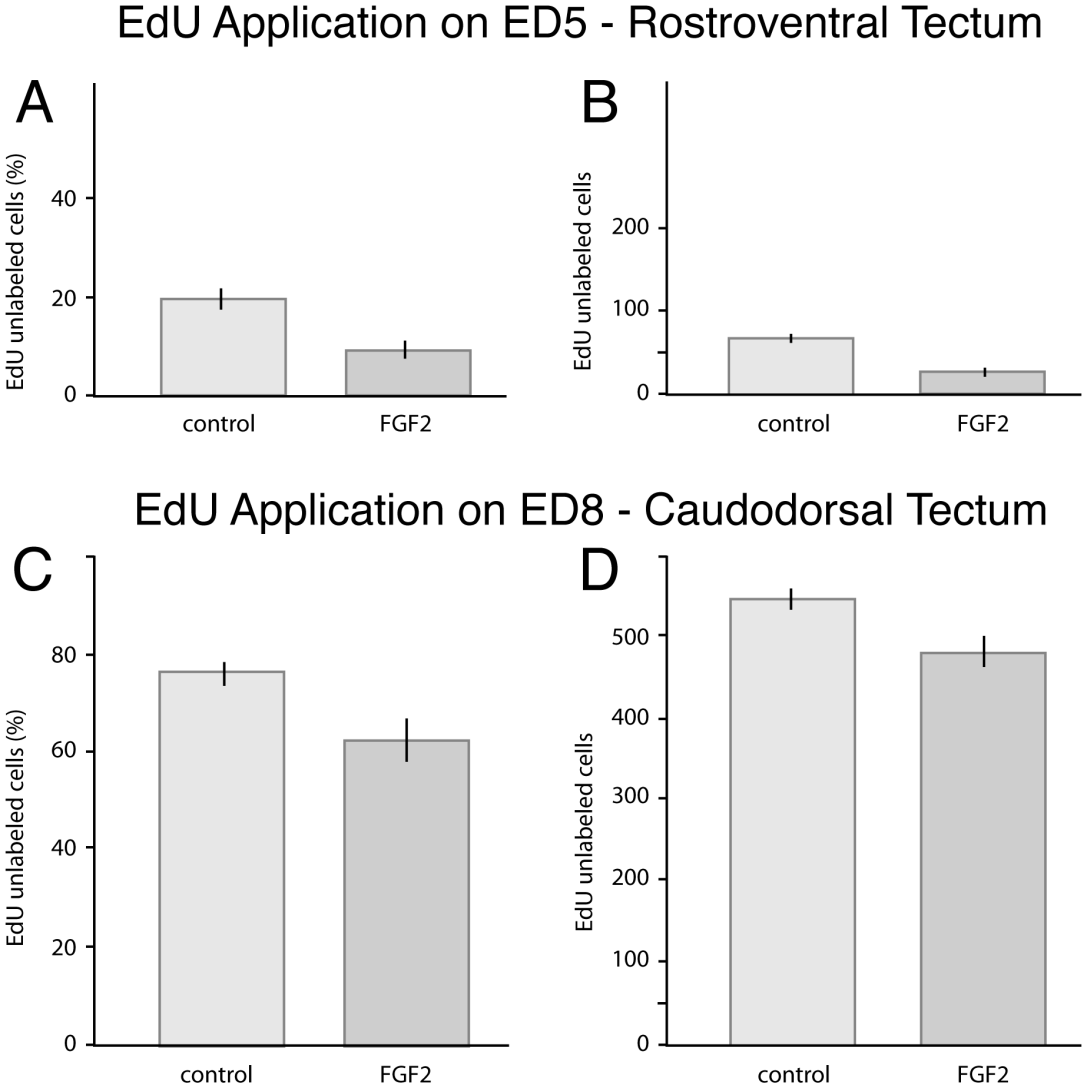
Quantitative data showing that FGF2 treatment delays tectal neurogenesis. In birds infused with EdU starting on ED5, the proportion of EdU-unlabeled cells in the early-born rostroventral tectum of FGF2-treated embryos is decreased by 52% relative to controls (A); the absolute number of EdU-unlabeled cells is decreased by 57% (B). Both observations are consistent with a delay in tectal neurogenesis in FGF2-treated birds. In birds infused with EdU starting on ED8, the proportion and absolute number of EdU-unlabelled cells in the caudodorsal tectum are decreased in the FGF2-treated embryos by 18% (C) and 12% (D). Again, both findings are consistent with a delay in neurogenesis. All mentioned differences are statistically significant (see text).

To determine whether FGF2 delays the onset of tectal neurogenesis, we began cumulative EdU-labeling in one group of embryos on ED5, 24 hours after FGF2 injection, and focused our analysis on the rostroventral tectum. When these embryos were sacrificed on ED12, the proportion of EdU-unlabeled cells in the rostroventral tectum was 52% lower in FGF2 treated birds than in controls (Figs. 4A,B, 5A; t(8) = 4.52; p<0.005; n = 5 controls, 5 FGF2-treated). Since the proportion of unlabeled cells could be affected by a change in the absolute number of EdU-labeled cells (as well as EdU-unlabeled cells), we quantified the absolute number of EdU-unlabeled cells per counting box. This number decreased by 57% in FGF2-treated embryos, relative to controls (Fig. 5B; t(8) = −6.80; p<0.0005; n = 5, 5). Both these findings are consistent with a delay in the onset of tectal neurogenesis in the rostroventral tectum of FGF2-treated birds.

### FGF2 treatment delays the end of tectal neurogenesis

To test whether FGF2 delays the end of tectal neurogenesis, as well as its beginning, we conducted a second set of experiments, in which we began the cumulative EdU-labeling on ED8, 4 days after the FGF2 treatment, and looked for changes in EdU labeling in the caudodorsal tectum. We found that the proportion and absolute numbers of EdU-unlabeled cells in the caudodorsal tectum decreased by 18% and 12%, respectively (Figs. 4C,D, 5C,D; proportion: t(9) = 3.03; p<0.01; n = 5, 6; absolute number: t(9) = −2.81; p<0.05; n = 5, 6). Both shifts are consistent with an FGF2-induced delay in the offset of neurogenesis in the caudodorsal tectum, though the effect is weaker than in the rostroventral tectum.

### FGF2 alters the morphology of some tectal layers

**Figure 6:**
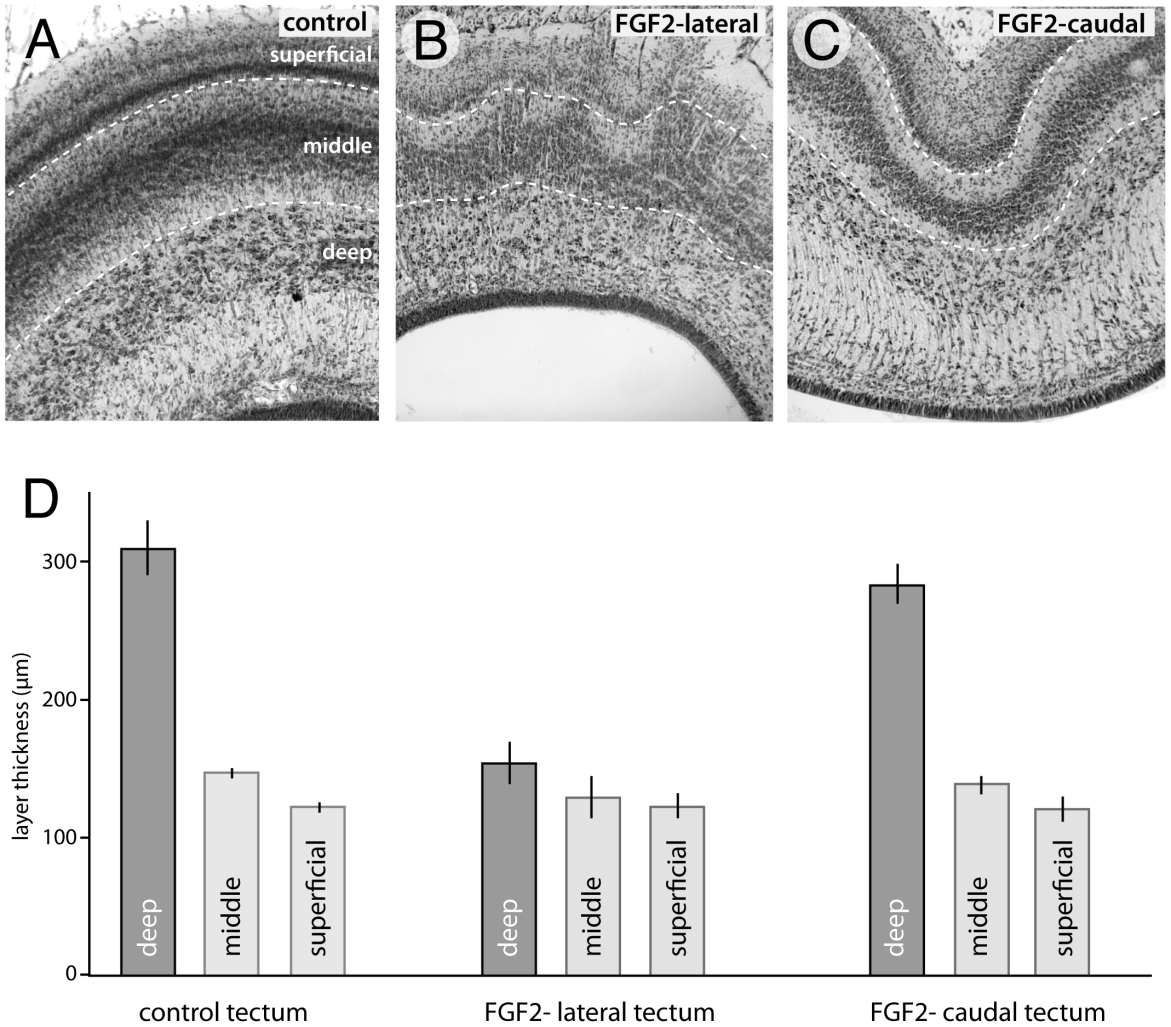
FGF2 decreases the thickness of early born tectal layers. By ED12, birds treated with FGF2 showed a 50% decrease in the radial thickness of the deep layers in the lateral tectum compared to controls (A, B, D). In these same lateral areas, despite the presence of “volcano”-like disturbances in the outer laminae (B), there is no statistically significant difference in the thickness of the superficial or the middle layers between FGF2-treated birds and controls (D). The caudal, folded areas of FGF2 treated birds (C) exhibited no significant changes in the thickness of any layers (D).

Since tectal laminae are born at different, though overlapping, times during development, one might expect FGF2-induced delays in neurogenesis to alter the thickness of some tectal laminae. Indeed, birds treated with FGF2 by ED12 showed a 50% decrease in thickness of the deep layers in the lateral tectum compared to controls (Fig. 6A, B, D; t(9) = −6.26; p<0.0005; n = 5, 6). There were no statistically significant group differences in the thickness of the superficial or middle laminae (Fig. 6D). The caudal, folded regions of the tectum exhibited no statistically significant group differences in any layers.

The observation that the superficial and middle tectal laminae were not thinned by the FGF2 treatment indicates that the FGF2 did not simply expand the mesencephalic ventricle without altering tectum volume, in which case it would have stretched all tectal laminae, making all of them thinner. Instead, it seems more likely that the FGF2-induced addition of more tectal cells drives a tangential (but not radial) expansion of some tectal laminae, which then expands the tectum's surface area. This hypothesis assumes that cell density within a laminae is not affected by FGF2.

To test the latter hypothesis, we counted cells within 100 µm-wide sampling regions in individual tectal laminae (see Methods). We observed that cell numbers in the deep tectal layers of the lateral tectum decreased by 39% (t(19) = −4.29; P<0.0005; n = 10, 11) in FGF2 treated birds relative to controls. The superficial layers showed a 24% decrease in cell number (t(19) = −2.32; P<0.05; n = 10, 11); whereas the middle layers showed no significant FGF2 effect. Collectively, these data indicate that the deep tectal layers were more severely affected by the FGF2 treatment than the other layers. Specifically, the deep tectal layers were thinner than normal and contained fewer cells per unit of tectal surface, whereas the superficial layers exhibited a slight decrease in cell numbers but retained their normal thickness.

In the caudal tectum the deep layers exhibited only an 11% decrease in cell numbers per counting box (t(19) = −2.34; P<0.05; n = 10, 11) in FGF2 treated birds relative to controls. There were no significant changes in cell number within the superficial or middle layers (t(19) = −0.24; P = 0.41; n = 10, 11). Thus, FGF2 has less effect on laminar thickness and cell numbers in the caudal tectum, where the expanded tectum folds (Fig. 6), than in more rostral regions.

### FGF2 alters the birthdates of some tectal layers

**Figure 7:**
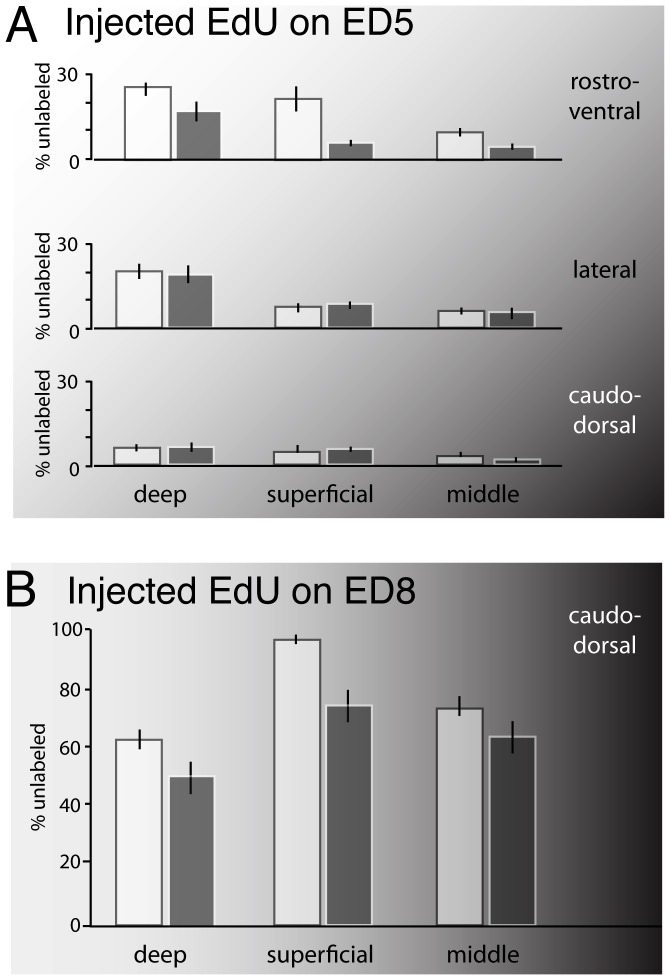
FGF2 delays the birthdates of neurons in deep, superficial and middle layers. In the rostroventral tectum of FGF2- treated embryos infused with EdU starting on ED5, the proportion of EdU-unlabeled cells is decreased by 32, 72, and 57% in the deep, superficial, and middle tectal layers, respectively (A). There were no statistically significant differences between FGF2 treated birds and controls in any layers in the lateral or caudal tectum when EdU was infused on ED5 (A). In embryos that were infused with EdU starting on ED8, the proportion of EdU-unlabeled cells was decreased by 19, 22, and 14% in the deep, superficial, and middle layers, respectively (B). White bars are controls; gray bars are FGF2 treated. Background shading indicates birth order gradients, with darker shades indicating later birthdates.

To test whether FGF2 changes the neuronal birthdates of tectal laminae we counted the proportion of EdU-unlabeled cells in the major layers. In birds infused with EdU starting on ED5, the proportion of EdU unlabeled cells in the deep layers of the rostroventral tectum was decreased by 32% in FGF2 treated birds relative to controls (Fig. 7A; t(8) = 1.96; P<0.05; n = 5, 5). In the superficial layers the proportion of EdU-unlabeled cells was decreased by 72% (Fig. 7A; t(8) = 3.61; P<0.01; n = 5, 5), and in the middle layers it decreased by 57% (Fig. 7A; t(8) = 3.29; P<0.01; n = 5, 5). These data indicate a significant delay in the average birthdates of the neurons in all tectal laminae, though the effect was weakest in the deep layers.

In birds infused with EdU starting on ED8, near the end of tectal neurogenesis, the proportion of EdU-unlabeled cells in the deep layers of the caudodorsal tectum was decreased by 19% in FGF2 treated birds relative to controls (Fig. 7B; t(9) = 2.02; P<0.05; n = 5, 6). The proportion of EdU-unlabeled cells was decreased by 22% in the superficial layers (t(9) = 4.53; P<0.005; n = 5, 6) and by 14% in the middle layers (t(9) = 1.51; P<0.05; n = 5, 6). These data, too, are consistent with delays in the birthdates of the average neurons in all laminae.

## Discussion

Several previous studies have shown that evolutionary increases in brain region volumes may result from delayed neurogenesis, which extends the number of cell divisions that progenitors may undergo [1], [2]. Consistent with such findings, researchers using experimental manipulations of proliferation have shown that delaying cell cycle exit is an effective way of increasing brain volume. For example, transgenic mice expressing a constitutively active form of beta-catenin in the neocortex exhibit delayed cortical neurogenesis as well as a dramatically enlarged neocortex [11].

Although a multitude of different molecules can regulate cell cycle exit, fibroblast growth factors (FGFs) are clearly critical players. Much of the relevant work has been performed on mammalian cells *in vitro* [12]–[14], but *in vivo* studies have confirmed that interfering with FGF signaling leads to premature cell cycle exit in mouse ventral midbrain/hindbrain [15]. Conversely, activation of FGF receptor 3 increases the size of occipital cortex in mice [16]. Most relevant to the present study, intra-ventricular injections of FGF2 in rats delay neocortical cell cycle exit in mouse neocortex, leading to dramatic increases cortex volume and neuron number [4]. A similar study in mice revealed a more localized increase in cortical proliferation, accompanied by the induction of a cortical fold [17]. Although these data indicate that FGF2 can regulate neurogenesis timing [18], FGF2 has also been shown to have additional functions, such as influencing whether postmitotic cells become neurons or glial cells [19].

Very little is known about the functions of FGF2 in birds. One study showed that FGF2 is present in the embryonic CSF of chicks and greatly enhances midbrain progenitor proliferation [20]. However, the reported effects suggest that FGF2 at those stages of development affects proliferation rates and/or progenitor survival, rather than neurogenesis timing. Our own previous study, in which we injected FGF2 into the ventricles of embryonic chicks, revealed a reduction in the relative thickness of the postproliferative zone in the optic tectum [3]. This finding is consistent with a delay in neurogenesis, but the evidence was indirect. In addition, our previous study provided no data on how long after the injection FGF2 can alter neurogenesis timing. It also did not show whether FGF increases the total number of tectal cells, and did not examine changes in laminar thickness or birthdates. The present paper provides all this information.

To test for an FGF2 induced delay of tectal neurogenesis, we focused our analysis on the early-born rostroventral tectum in embryos that were infused with EdU shortly after the beginning of tectal neurogenesis (ED5), and on the late-born caudodorsal tectum in embryos infused with EdU near the end of tectal neurogenesis (ED8). In both cases, a delay in cell cycle exit should manifest as an increase in the proportion of EdU-unlabeled cells (Fig. 2). Indeed, we found that FGF2 treatment decreased the proportion of EdU-unlabeled cells in both the rostroventral and caudodorsal tectum (in the ED5 and ED8 conditions, respectively; Fig. 5). Given that EdU-unlabeled cells must have been born before EdU application, the best explanation of our results is that FGF2 delayed cell cycle exit in tectal progenitors. The observed effect cannot be explained by an increase in tectal progenitor proliferation rates, because such an increase would, by itself, lead to an increase in EdU-labeled cells, rather than a decrease.

Because a delay in cell cycle exit was evident in both the rostroventral and caudodorsal areas, we conclude that FGF2 is acting on much of the tectum, rather than focally. This finding is important because it shows that FGF2 has an effect throughout the tectum. A previous studies had shown that, of the three FGF receptors likely to interact with FGF2 inside the brain, only FGFR1 is expressed uniformly in the tectum [21]. In contrast, FGFR2 and FGFR3 are expressed mainly in the rostral tectum, raising the possibility that FGF2 acts only on rostral tectum. Our finding of a neurogenesis delay in caudal tectum goes against that hypothesis, but the data do not exclude the possibility that FGF2 has a greater effect in the rostral tectum because of heterogeneous receptor expression.

In one set of experiments we injected FGF2 on ED4 and observed a delay in neurogenesis 4 days later, on ED8. This observation implies that FGF2 acted not just immediately after the injection on ED4, but for several days afterwards. Our observation that the FGF2-induced decrease in the number of EdU-unlabeled cells is greater when EdU is infused on ED5 than when it is infused on ED8 suggests that the potency of the injected FGF2 wanes over time, as one might expect, given that the injected FGF2 is never replenished. Alternatively, it may stem from a lower concentration of FGF2-binding receptors in the caudodorsal tectum, where we did our measurements in the embryos injected with EdU on ED8. Additional experiments are needed to disentangle these hypotheses.

If FGF2 delays tectal neurogenesis, then one would expect FGF2 to increase the number of cells within the tectum dramatically. Indeed, the cell counts in the present study, in conjunction with the earlier data on tectal surface area expansion [3], allow us to infer that FGF2 increases the total number of tectal cells by 140% (Fig. 3). Overall, these findings indicate that FGF receptors regulate neurogenesis timing not only in the mammalian telencephalon and ventral midbrain [4], [15], [18], but also in the avian optic tectum.

Given our findings, one may ask how a delay of tectal neurogenesis affects the birthdates of individual tectal layers, which are normally born at different, though overlapping stages of development [5]. In essence, our data indicate that FGF2 treatment delays average neuronal birthdates in all three major groups of laminae. Because these layers are born over the course of several days, our data support our hypothesis that the FGF2 effect is not transient but persistent, decreasing the probability of progenitor cell cycle exit for several days.

Even more interesting is that FGF2 treatment reduces cell density and laminar thickness most drastically in the deep layers, mildly in the superficial layers, and least in the middle layers. This pattern parallels the birthdate data obtained by Lavail and Cowan [5], who showed that the deep layers are born first, the middle layers last. Combining all this information, we can infer that the FGF2-induced delay of tectal neurogenesis promotes the production of late-born neurons at the expense of early-born neurons. This finding is consistent with comparative mammalian data indicating that evolutionary delays in neurogenesis generally increase the number of late-born cells at the expense of those born earlier ([2], [8], [22]. The general implication of these findings is that the timing of cell cycle exit can be dissociated from the mechanisms that control the timing of cell type specification (but see [7]). Unfortunately, the mechanisms that control cell fate in the nervous system remain relatively incompletely understood, even in mammalian neocortex, where they have been studied intensively (e.g., [23]). Still, our data support the hypothesis that the control of timing in development involves multiple, dissociable “clocks” [e.g., 24] and suggest that the chick optic tectum may be a good model system in which to explore this notion further.

## References

[1] Finlay BL, Darlington RB, Nicastro N (2001) Developmental structure in brain evolution. Behav Brain Sci 24: 263–278. 11530543

[2] Charvet CJ, Striedter GF, Finlay BL (2011) Evo-devo and brain scaling: candidate developmental mechanisms for variation and constancy in vertebrate brain evolution. Brain Behav Evol 78: 248–257. 21860220 10.1159/000329851 PMC3221253

[3] McGowan LD, Alaama RA, Freise AC, Huang JC, Charvet CJ, Striedter GF (2012) Expansion, folding, and abnormal lamination of the chick optic tectum after intraventricular injections of FGF2. Proc Natl Acad Sci USA 109: 10640–10646. 22723357 10.1073/pnas.1201875109 PMC3386876

[4] Vaccarino FM, Schwartz ML, Raballo R, Nilsen J, Rhee J, et al (1999) Changes in cerebral cortex size are governed by fibroblast growth factor during embryogenesis. Nat Neurosci 2: 246–253. 10195217 10.1038/6350

[5] LaVail JH, Cowan WM (1971) The development of the chick optic tectum: II. Autoradiographic studies. Brain Res 28: 421–441. 5111721

[6] Fujita S (1964) Analysis of neuron differentiation in central nervous system by tritiated thymidine autoradiography. J Comp Neurol 122: 311–327. 14184856 10.1002/cne.901220303

[7] Tarui T, Takahashi T, Nowakowski RS, Hayes NL, Bhide PG, et al (2005) Overexpression of p27 Kip 1, probability of cell cycle exit, and laminar destination of neocortical neurons. Cereb Cortex 15: 1343–1355. 15647527 10.1093/cercor/bhi017

[8] Finlay BL (2008) The developing and evolving retina: using time to organize form. Brain Res 1192: 5–16. 17692298 10.1016/j.brainres.2007.07.005

[9] Nishita J, Ohta S, Blyel SB, Schoenwolf GC (2011) Detection of isoform-specific fibroblast growth factor receptors by whole-mount in situ hybridization in early chick embryos. Dev Dyn 240: 1537–1547. 21465617 10.1002/dvdy.22616 PMC3092825

[10] Warren M, Puskarczyk K, Chapman SC (2009) Chick embryo proliferation studies using EdU labeling. Dev Dyn 238: 944–949. 19253396 10.1002/dvdy.21895 PMC2664394

[11] Chenn A, Walsh CA (2002) Regulation of cerebral cortical size by control of cell cycle exit in neural precursors. Science 297: 365–369. 12130776 10.1126/science.1074192

[12] Bouvier MM, Mytilineou C (1995) Basic fibroblast growth factor increases division and delays differentiation of dopamine precursors in vitro. J Neurosci 15: 7141–7149. 7472468 10.1523/JNEUROSCI.15-11-07141.1995 PMC6578057

[13] Deloulme JC, Gensburger C, Sarhan S, Seiler N, Sensenbrenner M (1991) Effects of basic fibroblast growth factor on the development of GABAergic neurons in culture. Neuroscience 42: 561–568. 1716750 10.1016/0306-4522(91)90398-8

[14] Vescovi AL, Reynolds BA, Fraser DD, Weiss S (1993) bFGF regulates the proliferative fate of unipotent (neuronal) and bipotent (neuronal/astroglial) EGF-generated CNS progenitor cells. Neuron 11: 951–966. 8240816 10.1016/0896-6273(93)90124-a

[15] Lahti L, Saarimäki-Vire J, Rita H, Partanen J (2011) FGF-signaling gradient maintains symmetrical proliferative divisions of midbrain neuronal progenitors. Dev Biol 349: 270–282. 21074523 10.1016/j.ydbio.2010.11.008

[16] Thomson RE, Kind PC, Graham NA, Etherson ML, Kennedy J, et al (2009) Fgf receptor 3 activation promotes selective growth and expansion of occipitotemporal cortex. Neural Dev 4: 4. 19192266 10.1186/1749-8104-4-4 PMC2661882

[17] Rash BG, Tomasi S, Lim HD, Suh CY, Vaccarino FM (2013) Cortical gyri- fication induced by fibroblast growth factor 2 in the mouse brain. J Neurosci 33: 10802–10814. 23804101 10.1523/JNEUROSCI.3621-12.2013 PMC3693057

[18] Rash BG, Lim HD, Breunig JJ, Vaccarino FM (2011) FGF signaling expands embryonic cortical surface area by regulating Notch-dependent neurogenesis. J Neurosci 31: 15604–15617. 22031906 10.1523/JNEUROSCI.4439-11.2011 PMC3235689

[19] Yoon K, Nery S, Rutlin ML, Radtke F, Fishell G, Gaiano N (2004) Fibroblast growth factor receptor signaling promotes radial glial identity and interacts with Notch1 signaling in telencephalic progenitors. J Neurosci 24: 9497–9506. 15509736 10.1523/JNEUROSCI.0993-04.2004 PMC6730142

[20] Martín C, Bueno D, Alonso MI, Moro JA, Callejo S, et al (2006) FGF2 plays a key role in embryonic cerebrospinal fluid trophic properties over chick embryo neuroepithelial stem cells. Dev Biol 297: 402–416. 16916506 10.1016/j.ydbio.2006.05.010

[21] Walshe J, Mason I (2000) Expression of FGFR1, FGFR2 and FGFR3 during early neural development in the chick embryo. Mech Dev 90: 103–110. 10585567 10.1016/s0925-4773(99)00225-7

[22] Rapaport DH, Rakic,P, LaVail MM (1996) Spatiotemporal gradients of cell genesis in the primate retina. Perspect Dev Neurobiol 3: 147–159. 8931090

[23] Desai AR, McConnell SK (2000) Progressive restriction in fate potential by neural progenitors during cerebral cortical development. Development 127: 2863–2872. 10851131 10.1242/dev.127.13.2863

[24] Zhang L, Kendrick C, Jülich D, Holley SA (2008) Cell cycle progression is required for zebrafish somite morphogenesis but not segmentation clock function. Development 135: 2065–2070. 18480162 10.1242/dev.022673 PMC2923836

